# Selective Paracrine Modulation of Stromal Cells: Wharton’s Jelly MSC Secretome Enhances Adipose-Derived MSC Functionality While Maintaining Dermal Fibroblast Quiescence

**DOI:** 10.3390/ijms262010095

**Published:** 2025-10-16

**Authors:** Tanya Stoyanova, Lora Topalova, Stanimir Kyurkchiev, Regina Komsa-Penkova, Svetla Todinova, George Altankov

**Affiliations:** 1 Institute of Biophysics and Biomedical Engineering, Bulgarian Academy of Sciences, 1113 Sofia, Bulgaria; tanya.zh.stoyanova@gmail.com (T.S.); topalovaloram@gmail.com (L.T.); todinova@abv.bg (S.T.); 2 Center of Competence in Personalized Medicine, 3D and Telemedicine, Robotic Assisted and Minimally Invasive Surgery—“Leonardo da Vinci”, 5800 Pleven, Bulgaria; regina.komsa-penkova@mu-pleven.bg; 3 Tissue Bank BulGen, 1330 Sofia, Bulgaria; stanimirkyurkchiev@gmail.com; 4 Department of Biochemistry, Medical University-Pleven, 5800 Pleven, Bulgaria; 5 Associate Project BG-RRP-2.004-0003, Medical University Pleven, 5800 Pleven, Bulgaria; 6 Research Institute, Medical University Pleven, 5800 Pleven, Bulgaria

**Keywords:** Wharton’s jelly MSCs, secretome, paracrine crosstalk, human adipose MSCs, primary fibroblasts, collagen interaction secretion, wound healing

## Abstract

Wharton’s jelly-derived mesenchymal stem cells (WJ-MSCs) secrete a rich array of paracrine factors including growth factors, cytokines, and extracellular vesicles that hold promises for regenerative medicine. This study evaluated the effects of WJ-MSC-derived secretome on adipose-derived mesenchymal stem cells (AD-MSCs) and human dermal fibroblasts (HDFs), focusing on their adhesion, spreading, proliferation, endogenous collagen secretion, and migration. Morphometric analysis revealed that the secretome enhanced cell adhesion and spreading on rat tail collagen (RTC) substrates after 24 h. AD-MSCs showed a ~30% increase in the cell spreading area (from 4007 μm^2^ to 5081 μm^2^ *p* < 0.05), though without notable shape changes. In contrast, fetal bovine serum (FBS) promoted cell elongation with a reduced aspect ratio. Proliferation assays demonstrated a selective stimulatory effect of the secretome on AD-MSCs with a significant increase at day 3, while HDFs’ proliferation remained unchanged. Cell cycle profiling showed transient S-phase accumulation in AD-MSCs (24–48 h), followed by G0/G1 arrest (72 h), while HDFs remained in G0/G1. Immunofluorescence analysis confirmed the enhanced extracellular deposition of endogenously synthesized collagen in AD-MSCs, while no comparable response was observed in HDFs. Scratch assays showed increased migration in both cell types upon secretome exposure compared to collagen-only controls, suggesting a paracrine-mediated pro-migratory effect. These results demonstrate that WJ-MSC secretome boosts the regenerative capacity in AD-MSCs while keeping fibroblasts quiescent, highlighting its strong potential for cell-free therapies in tissue engineering, wound repair, and regenerative medicine.

## 1. Introduction

Mesenchymal stem cells (MSCs) are multipotent stromal cells capable of self-renewal and differentiation into various mesodermal lineages, including osteoblasts, chondrocytes, and adipocytes. First isolated from bone marrow, MSCs have since been identified in numerous tissues, such as adipose tissue, placenta, and the umbilical cord, among others [1]. In the past decade, MSCs have emerged as a promising therapeutic modality in regenerative medicine due to their immunomodulatory properties, trophic support, and ability to hone in on sites of tissue injury. Although initially believed to contribute to tissue repair through direct differentiation and engraftment, accumulating evidence suggests that their primary mechanism of action is mediated via paracrine signaling [2,3,4]. Following systemic or local administration, MSCs exhibit a transient lifespan often limited to 48–72 h yet exert significant biological effects through the secretion of bioactive molecules, including cytokines, chemokines, growth factors, and extracellular vesicles such as exosomes [5]. These secreted factors, often termed secretome, play a pivotal role in modulating the local microenvironment, promoting angiogenesis, attenuating inflammation, and stimulating the activation and proliferation of endogenous stem and progenitor cells [3,6]. Consequently, the regenerative outcomes observed in MSC-based therapies are increasingly attributed to the indirect activation of host repair mechanisms rather than the direct cellular replacement by the transplanted MSCs themselves [4]. This paradigm shift underscores the importance of understanding the molecular composition and functional relevance of the MSC secretome in order to optimize therapeutic efficacy and design next-generation cell-free regenerative strategies.

Among the various MSC sources, Wharton’s jelly—the gelatinous connective tissue within the umbilical cord—has emerged as a particularly promising reservoir of MSCs, as they are easily accessible, ethically non-controversial, and exhibit a high proliferative capacity and low immunogenicity. Accumulating evidence suggests a unique therapeutic potential of Wharton’s jelly-derived mesenchymal stem cells (WJ-MSCs) [7,8,9], which have recently led to growing commercial interest. Several companies now offer cryopreserved WJ-MSCs collected at birth, with the intention of future therapeutic use for the donor child [10,11,12]. Emerging research, however, increasingly indicates that the actual therapeutic efficacy of Wharton’s jelly MSCs is similarly attributed to their paracrine activity—namely, the bioactive molecules they secrete collectively rather than through direct cellular engraftment or replacement [7,13,14]. Indeed, Wharton’s jelly MSC secretome encompasses a complex mixture of soluble proteins, cytokines, chemokines, growth factors, and extracellular vesicles (EVs), including exosomes and microvesicles (Trigo et al., 2025 [15,16]). These bioactive components modulate inflammation, promote angiogenesis, and support tissue repair and regeneration. Though the composition and potency of the secretome vary depending on the MSC source, WJ-MSCs demonstrate a particularly rich and functionally diverse profile [17].

As interest in cell-free regenerative therapies continues to accelerate, the secretome derived from WJ-MSCs has gained recognition as a promising, scalable, and low-risk alternative to direct cell transplantation [7]. While its therapeutic potential is increasingly acknowledged, the ability of the WJ-MSC secretome to influence the behavior of other mesenchymal stem cell populations such as adipose-derived MSCs (AD-MSCs) remain insufficiently explored. Likewise, its effects on other adult cell types, particularly fibroblasts, which are key players in tissue repair and remodeling, are still poorly understood [18]. Indeed, recent advances have reshaped our understanding of MSC interactions with fibroblasts, particularly in the context of fibrosis. A pivotal study by Xu and colleagues (2023) [19] demonstrated that MSCs can reversibly de-differentiate myofibroblasts back into fibroblast-like cells by selectively inhibiting the transforming growth factor-β/small body size genes and mothers against the decapentaplegic genes 2/3 (TGF-β/SMAD2/3) signaling pathway, a central axis in fibrotic progression. This finding challenges the long-held notion that myofibroblasts are terminally differentiated and opens new therapeutic avenues for treating fibrotic diseases such as idiopathic pulmonary fibrosis and bronchiolitis obliterans. Importantly, Xu et al. (2023) [19] revealed that these de-differentiated fibroblast-like cells retain sensitivity to the transforming growth factor-β 1 (TGF-β1) and can re-enter the myofibroblast state if the pro-fibrotic microenvironment persists. This underscores the need for sustained modulation of the niche and highlights the dynamic plasticity of fibroblast phenotypes under MSC influence. Similar observations were made by Dyachkova and colleagues (2023) [20], who showed that chronic inflammation mediated by M2 macrophages can induce reversible senescence in MSCs, thereby affecting their anti-fibrotic properties. These insights deepen our mechanistic understanding of MSC–fibroblast crosstalk and provide a rationale for the variable clinical outcomes observed in MSC-based therapies for fibrotic conditions. Earlier work by Desai, Hsia, and Schwarzbauer (2014) [21] also supports the concept of reversible modulation of myofibroblast differentiation, demonstrating that adipose-derived MSCs can influence the fibroblast phenotype in a context-dependent manner. Together, these studies highlight the therapeutic potential of MSCs not only as regenerative agents but also as dynamic modulators of fibrotic remodeling.

A key question arises in the context of cell-free regenerative therapies: are there significant differences between the secretomes of WJ-MSCs and AD-MSCs? Comparative studies have indeed revealed distinct variations in their secretory profiles. Proteomic analyses show that WJ-MSCs secrete a broader and more potent array of bioactive molecules, including high levels of immunomodulatory cytokines such as interleukin 10 (IL-10), transforming growth factor-β (TGF-β)**,** and hepatocyte growth factor (HGF), as well as pro-regenerative factors like vascular endothelial growth factor (VEGF), insulin-like growth factor 1 (IGF-1), and fibroblast growth factor 2 (FGF-2). In contrast, AD-MSCs tend to produce a more tissue-specific secretome with relatively higher concentrations of interleukin 6 (IL-6), membrane cofactor protein 1 (MCP-1), and matrix metalloproteinases (MMPs), which support localized repair but offer limited systemic paracrine signaling [7,13,17,22]. Mechanistically, the regenerative potential of WJ-MSCs shall be largely attributed to their paracrine activity, involving the secretion of the above key bioactive molecules. These factors play crucial roles in angiogenesis, fibroblast recruitment, extracellular matrix (ECM) remodeling, and immunomodulation [7,22]. VEGF promotes endothelial cell proliferation and neovascularization, platelet-derived growth factor (PDGF) supports mesenchymal cell migration and matrix deposition, while TGF-β is central to wound healing and fibrosis regulation. The coordinated release of these molecules by WJ-MSCs underscores their superior paracrine signaling capacity and positions them as a promising cell-free therapeutic source.

These molecular distinctions are particularly relevant for regenerative medicine, as the composition of the secretome can critically influence therapeutic efficacy. WJ-MSCs, with their enriched profile of anti-inflammatory and angiogenic factors, may be better suited for treating systemic inflammatory conditions and promoting tissue regeneration across diverse organ systems. Meanwhile, AD-MSCs may offer advantages in targeted therapies where localized tissue remodeling is desired.

Equally underexplored is the role of the surrounding ECM, especially collagen, the primary structural protein within it. Collagen contributes to tissue regeneration not only by offering mechanical support but also by delivering essential biochemical signals that facilitate effective cellular interactions. In stem cell biology, collagen enhances adhesion, survival, and proliferation, while also influencing differentiation pathways through integrin-mediated and other signaling mechanisms [23,24,25]. All types of MSCs are particularly responsive to collagen-rich microenvironments, which promote better engraftment and drive lineage-specific differentiation—most notably toward osteogenic, chondrogenic, and adipogenic outcomes [26]. Furthermore, collagen serves as a bioactive scaffold that closely replicates the native ECM, facilitating key cellular processes such as stem cell migration, spatial organization, and tissue remodeling, essential for successful regenerative therapies [27,28,29]. These regenerative dynamics can be effectively assessed in vitro using the artificial wound healing (scratch) assay—a widely accepted technique for quantifying cell migration and matrix remodeling [30]. In this study, we applied the scratch assay to investigate the influence of WJ-MSC secretome on the migratory behavior of both AD-MSCs and fibroblasts cultured on collagen-coated substrates. This builds upon our previous methodological framework [25,31], which demonstrated the MSC-mediated remodeling of adsorbed collagen under oxidative stress or high glucose conditions using both morphological and morphometric analyses. Together, these approaches highlight the responsiveness of MSCs to microenvironmental cues and underscore their relevance in modeling pathological tissue repair. They also show that collagen is not merely a passive scaffold; it undergoes active remodeling through interactions between stem cells and fibroblasts, contributing to its dynamic turnover within tissues. This remodeling is particularly pronounced during wound healing and regeneration, where collagen serves not only as a structural matrix but also as a signaling platform that orchestrates new tissue formation [32,33].

This study aimed to systematically evaluate the effects of the WJ-MSC secretome on the key functional parameters of AD-MSCs and in a comparative plan on primary human fibroblasts. Emphasis was placed on cell adhesion to adsorbed rat tail collagen (RTC), distinctly assessed from the intracellular collagen by using species-specific antibodies visualized through immunofluorescence. Additional endpoints included a quantitative analysis of cell morphology, proliferative activity, and cell cycle dynamics of both cell types, alongside an assessment of their migratory behavior using an in vitro scratch wound assay.

## 2. Results

### 2.1. Effect of the WJ-MSCs’ Secretome on the Morphology of AD-MSCs and Fibroblasts

Aligned with the central aim of this study, we initially explored the impact of the WJ-MSCs on the adhesion and morphological characteristics of adipose-derived mesenchymal stem cells (AD-MSCs) and human dermal fibroblasts (HDFs), all originating from distinct mesenchymal tissues. Collagen was selected as the substrate protein due to its prominent role as a key ECM component, essential for tissue regeneration and the whole stem cell biology field [26].

**Figure 1:**
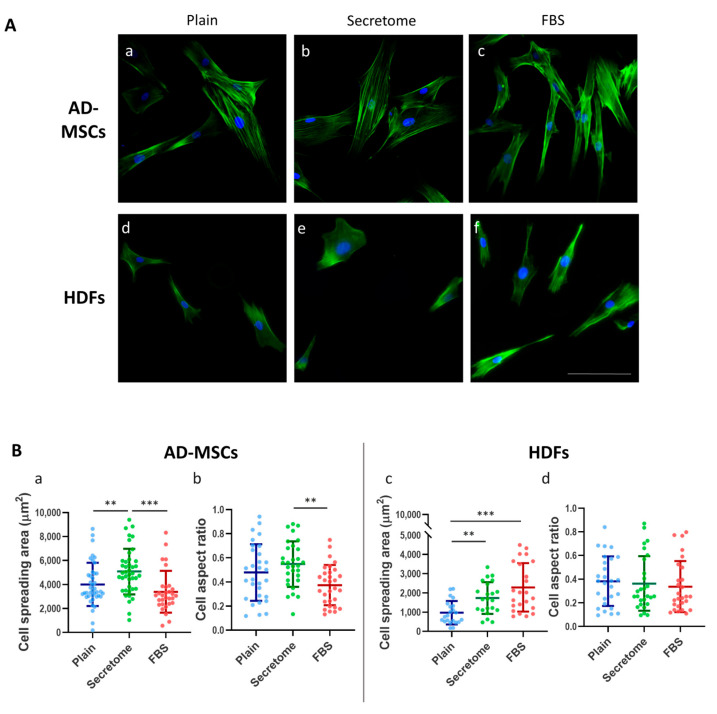
Wharton’s jelly-derived mesenchymal stem cells (WJ-MSCs) secretome affects the morphology of adipose tissue derived MSCs and HDFs adhering on collagen-coated substrates. (**A**): Representative images of adipose-derived mesenchymal stem cells (AD-MSCs) (**a**–**c**) and human dermal fibroblasts (HDFs) (**d**–**f**) cultured for 24 h on collagen-coated surfaces under different conditions, namely, (**a**,**d**) untreated control cells (plain); (**b**,**e**) cells cultured in the presence of WJ-MSC-derived secretome; and (**c**,**f**) cells cultured in the presence of 10% fetal bovine serum (FBS) (positive control). Cells were stained with phalloidin (green) to visualize F-actin and Hoechst (blue) for nuclear DNA. (**B**): Quantitative morphometric analysis of cell morphology including cell spreading area and aspect ratio of AD-MSC (**a**,**b**) and HDF (**c**,**d**). The data are presented as mean ± SEM. Asterix denotes statistical significance: *p*  <  0.01 (**), and *p*  <  0.001 (***). Scale bar in (**A**): 50 μm.

As depicted in Figure 1A, exposure to the WJ-MSC-derived secretome administered at a concentration equivalent to that present in the native WJ-MSC culture medium resulted in the enhanced cell spreading of both AD-MSCs and HDFs after 24 h of incubation (Figure 1A(b) and Figure 1A(e), respectively) compared to cells cultured on collagen-only substrates (Figure 1A(a,d)). Notable differences in cytoskeletal architecture were also observed. For AD-MSC samples, incubation with secretome led to the development of prominent actin stress fibers, indicating enhanced cytoskeletal organization. Conversely, samples treated with FBS showed less pronounced stress fiber formation, characterized by a primarily cortical arrangement, while cells grown on plain collagen revealed an intermediate cytoskeletal phenotype (Figure 1A(a–c)). Fibroblasts, in the same conditions, exhibited less developed actin networks compared to AD-MSCs, while treatment with FBS induced a more polarized morphology in both cell types (Figure 1A(c,f)), evident by the elongated cell shape, the corresponding increase in the cell aspect ratio (AR), and directionally oriented actin stress fibers. These results suggest the differential modulation of cytoskeletal dynamics depending on the bioactive environment. In this respect, it is important to note that these morphological characteristics were assessed after 24 h of incubation—a time point where the organization of the actin cytoskeleton differs significantly from the early stages of cell adhesion.

The quantitative morphometric analysis shown on Figure 1B and Table 1 revealed statistically significant differences in the cell spreading area (CSA) and AR for both AD-MSCs and HDFs (Figure 1B). In the presence of the WJ-MSC secretome, AD-MSCs exhibited a ~30% increase in the CSA, expanding from 4007 μm^2^ on unmodified collagen to 5081 μm^2^ (Figure 1B(a)), indicative of an enhanced cell–substrate interaction. This expansion was accompanied by an approximate 15% increase in AR, suggestive of a slightly diminished cellular polarization. Conversely, under serum-enriched conditions (the positive control), AD-MSCs demonstrated a ~15% reduction in the CSA, decreasing to 3389 μm^2^ from the baseline of 4007 μm^2^ (Figure 1B(a)), which was correlated with a concomitant decrease in AR values (Figure 1B(b); Table 1), implying increased cellular elongation and potential directional migration.

Fibroblasts, typically over threefold smaller in size compared to AD-MSCs, also demonstrated enhanced spreading in the presence of the WJ-MSC secretome, with the CSA surprisingly increasing by approximately 79% from 971.6 μm^2^ to 1737 μm^2^ (Figure 1B(c)). The increased values are presented in % because of the initial difference in cell size between WJ-MSC and fibroblasts. However, despite this improvement, their CSA remained intermediate and did not reach the values observed in fibroblasts cultured with FBS, where a CSA of 2289 μm^2^ was recorded (corresponding to a 135.6% increase). AR values for fibroblasts remained relatively stable at around 0.350 (Figure 1B(d), Table 1), showing only modest decreases ranging from 5% to 11.9% compared to controls, indicating a slight increase in cellular polarization (Figure 1A(e,f)).

### 2.2. WJ-MSC Secretome Supports the Growth of MSCs but Not of Fibroblasts

Building upon our preliminary findings that the WJ-MSC-derived secretome enhances the proliferation of AD-MSCs, we now provide a more comprehensive analysis of this effect, including a comparative evaluation of its influence on fibroblasts. This approach is motivated by the involvement of both cell types in tissue regeneration and the clear relevance of this experimental observation to regenerative medicine.

**Figure 2:**
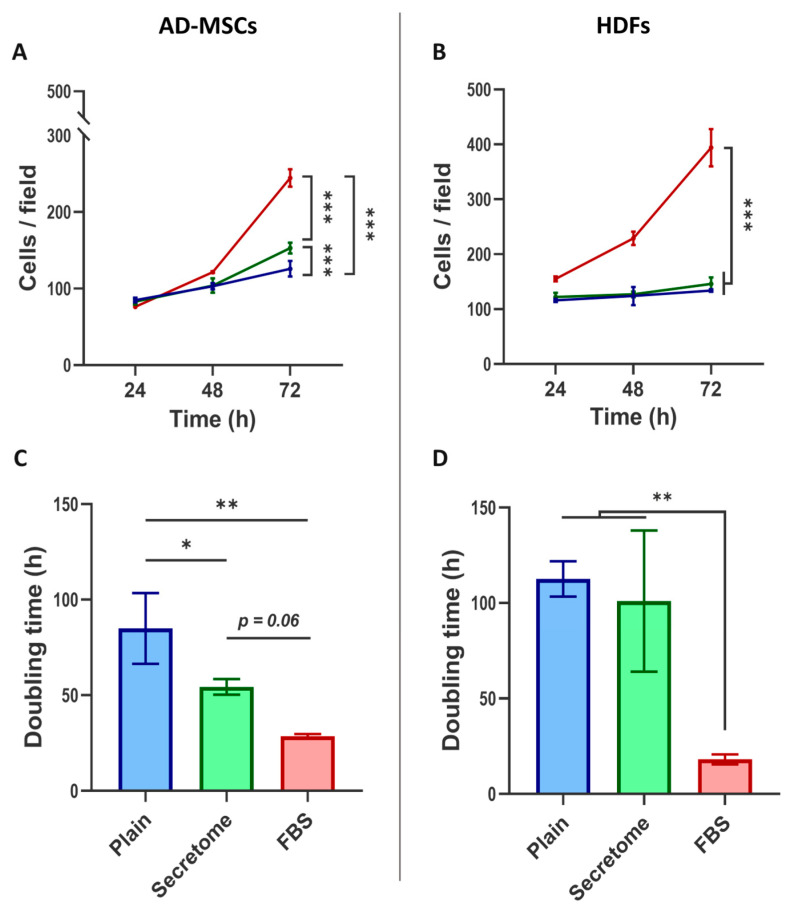
WJ-MSCs’ secretome enhances cell proliferation and reduces doubling time in AD-MSCs but has no effect on HDFs when cultured on collagen-coated surfaces. Graphs (**A**,**C**) show proliferation dynamics of AD-MSCs presented as cells per field, while (**B**,**D**) are of HDFs. The cells were cultured for 72 h on collagen-coated substrata in serum-free medium (blue) or medium supplemented with either WJ-MSC-derived secretome (green) without FBS or with 10% FBS (red). (**A**) The cell number was quantified at 24, 48, and 72 h (**A**,**B**), while cell doubling time was calculated over the full 72 h period (**C**,**D**). For graphs (**A**) and (**B**), statistical analysis was performed at the final time point (72 h). All data are presented as mean ± SEM. Asterix denotes statistical significance: *p*  <  0.05 (*), *p* < 0.01 (**), and *p* < 0.001 (***).

As shown in Figure 2A, the WJ-MSC-derived secretome promotes the proliferation of AD-MSCs cultured on collagen substrates under serum-free conditions, with a statistically significant effect observed by day 3.

In contrast, HDFs cultured under identical conditions did not exhibit a notable increase in cell number (Figure 2B), except in the presence of FBS, where cell counts were significantly elevated on both day 2 and day 3, reaching approximately double the control levels by day 3 for both cell types.

The influence of the secretome on cell proliferation kinetics is more clearly illustrated by analyzing the cell doubling time (Figure 2C,D). For AD-MSCs, secretome treatment resulted in a substantial reduction in the doubling time, approximately twofold compared to the controls (Figure 2C), again indicating an accelerated proliferation. However, no significant change in the cell doubling time was observed for HDFs under the same conditions (Figure 2D). In both cell types, FBS supplementation consistently yielded the shortest doubling times, confirming its potent mitogenic effect.

### 2.3. Impact of Paracrine Signaling from WJ-MSCs on Cell Cycle Progression in Stem Cells and Fibroblasts

**Figure 3:**
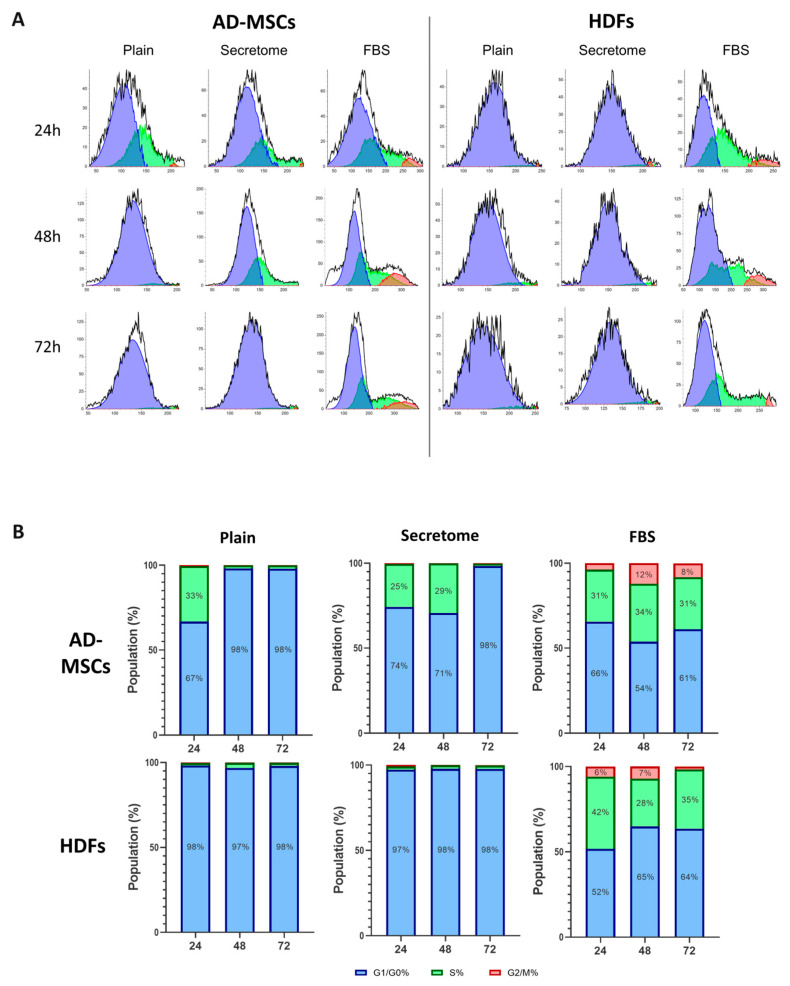
WJ-MSC secretome selectively modulates cell cycle progression in MSCs, but not in HDFs, cultured on collagen-coated substrates. Fluorocytometric analysis of nuclear DNA content was performed using Hoechst staining in AD-MSCs and HDFs cultured for 24, 48, and 72 h under three conditions: plain medium (control), medium supplemented with WJ-MSC-derived secretome (secretome), and medium supplemented with 10% FBS. (**A**) displays representative DNA histograms, where event count (y-axis) is plotted against total nuclear fluorescence intensity (x-axis), reflecting DNA content. Cell cycle phases are color-coded: G0/G1 (blue), S (green), and G2/M (red). AD-MSCs and HDFs were analyzed at each time point (24 h, 48 h, and 72 h), with conditions indicated as control (plain medium), secretome (WJ-MSC-derived secretome), and FBS (10% FBS supplementation). (**B**) presents quantitative analysis of cell cycle phase distribution, expressed as a percentage of the total cell population for AD-MSCs (upper row) and HDFs (lower row). Color coding is consistent across the two panels.

To better clarify the effect of WJ-MSCs’ secretome, we further performed an image-based fluorocytometric analysis of the nuclear DNA content, as viewed by Hoechst staining of the same AD-MSCs and HDF cells cultured for 24, 48, and 72 h (Figure 3). WJ-MSCs’ secretome treatment leads to faster proliferation in stem cells compared to the control with plain media, observable at 48 h and more so at 72 h after the start of the incubation, with a doubling time 1.6 times faster than that of the non-treated samples but still about 1.9 times slower than the cells incubated with FBS 10%. This tendency was not present when fibroblasts were treated with the secretome, where no difference was found in the proliferation rate.

As shown in Figure 3, the WJ-MSC-derived secretome exerts distinct modulatory effects on cell cycle progression in MSCs while having minimal impact on HDFs. MSCs exposed to the secretome under serum-free conditions display a transient accumulation in the S phase at 24 and 48 h, followed by a pronounced shift toward G0/G1 by 72 h (Figure 3B, upper row), indicative of entry into a quiescent state. In contrast, fibroblasts treated with the secretome remain consistently arrested in the G0/G1 phase throughout the entire observation period, with no detectable transition into S or G2/M phases (Figure 3B, lower row). These cell cycle profiles are consistent with the previously noted differences in cell proliferation and doubling time.

### 2.4. Paracrine Signaling from WJ-MSCs Modulates Extracellular Collagen Deposition

A key hallmark of mesenchymal cell function is the production and organization of the ECM [34], with collagen playing a central role. In this context, we investigate the influence of the WJ-MSC secretome on the intracellular processing and substratum deposition of collagen by AD-MSCs and HDFs, again in a comparative plan. Specifically, we examine the fate of the endogenous type I collagen produced by these cells using an anti-human collagen antibody while they are cultured on an RTC, which is not detected by the antibody.

**Figure 4:**
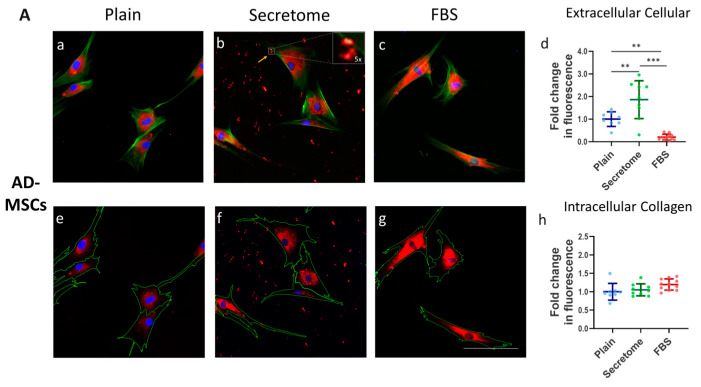

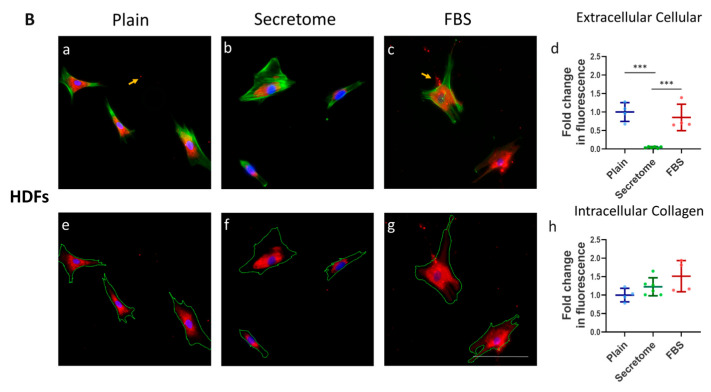
WJ-MSC secretome differentially regulates extracellular collagen deposition by MSCs and HDFs. Immunofluorescence imaging and quantitative analysis of collagen type I localization in AD-MSCs (**A**) and HDFs (**B**) cultured under three conditions: serum-free control (plain; images (**a**,**e**)), WJ-MSC-derived secretome (secretome; images (**b**,**f**)), and 10% FBS supplementation (FBS; images (c, g)). (**A**) (**a**–**c**) shows representative triple-stained images for nuclei (Hoechst, blue), F-actin (phalloidin, green), and collagen I (Alexa Fluor 555, red). Corresponding images (**e**–**g**) display collagen and nuclei only, with cell borders overlaid (based on cytoskeletal contours) to enable segmentation and quantification of intra- and extracellular collagen. Yellow arrows indicate extracellular collagen deposits. Inset in (**A**)(**b**) displays a 5× magnified view of secreted collagen, highlighting its clustered morphology. Quantitative analysis of extracellular collagen fluorescence, normalized to cell number, is shown in graphs (**A**)(**d**) for AD-MSCs and (**B**)(**d**) for HDFs. Intracellular collagen levels are presented in (**A**)(**h**) and (**B**)(**h**), expressed as fold change relative to control. Statistical significance is indicated as follows: *p*  <  0.01 (**) and *p*  <  0.001 (***). Scale bar: 100 μm.

Figure 4 illustrates the intracellular organization and extracellular deposition of endogenous human type I collagen following 24 h incubation of AD-MSCs (Panel A) and HDFs (Panel B) on RTC-coated substrata. The impact of the WJ-MSC secretome (images in Figure 4A(b,f),B(b,f)) is assessed relative to plain collagen-coated controls (Figure 4A(a,d),B(a,d)) and FBS supplementation (Figure 4A(c,g),B(c,g)), which serves as a positive control.

In both Figure 4A,B, the bottom row displays the same cells as on the top row but with outlined contours to delineate intracellular and extracellular compartments—an essential distinction for the subsequent microfluorimetric quantifications shown in diagrams (d and h) on both panels (Figure 4A,B), respectively.

An analysis of Figure 4A revealed that the WJ-MSC secretome significantly enhanced the extracellular deposition of collagen type I by AD-MSCs. This was evidenced by the clustered fluorescence signals (indicated by an arrow in image b on Figure 4A) and quantitatively supported by elevated fluorescence intensity (images d and h on Figure 4A). The deposited collagen appeared morphologically disorganized, forming dense clusters without clear spatial alignment (see the 5× augmented inset on image b). These differences were statistically significant, as shown in graph (Figure 4A(d)).

In contrast to AD-MSCs, HDFs cultured under identical conditions (Figure 4B) demonstrated significantly lower levels of extracellular collagen type I deposition in response to the WJ-MSC secretome (Figure 4B(b,f)). This reduction was evident when compared to both the plain collagen control (Figure 4B(a,e)) and the FBS-supplemented samples (Figure 4B(c,g)), with statistical significance confirmed in Figure 4B(d).

Additionally, intracellular collagen fluorescence in both cell types (Figure 4A,B) exhibited a consistent perinuclear localization. The fluorescence intensity remained intermediate between the plain collagen and FBS-supplemented conditions, with no statistically significant differences observed (Figure 4A(h),B(h)). This distribution pattern is morphologically indicative of Golgi-associated intracellular processing, where newly synthesized collagen undergoes post-translational modifications such as hydroxylation, glycosylation, etc., that ensure proper trimer assembly and trafficking [35,36].

### 2.5. WJ-MSC Secretome Enhances Migration of Both Stem Cells and Fibroblasts

Another essential hallmark of the regenerative potential of mesenchymal stem cells and fibroblasts is their capacity for directed migration toward sites of tissue injury—an ability that can be effectively simulated using the in vitro wound healing assay, commonly referred to as the scratch assay [37]. Figure 2 illustrates the impact of the WJ-MSC-derived secretome on the migratory behavior of AD-MSCs and HDFs, as evaluated through the time-lapse microscopy of live cells (see the Materials and Methods section). The figure presents only representative images captured at the initiation of the assay and following 24 h of incubation.

**Figure 5:**
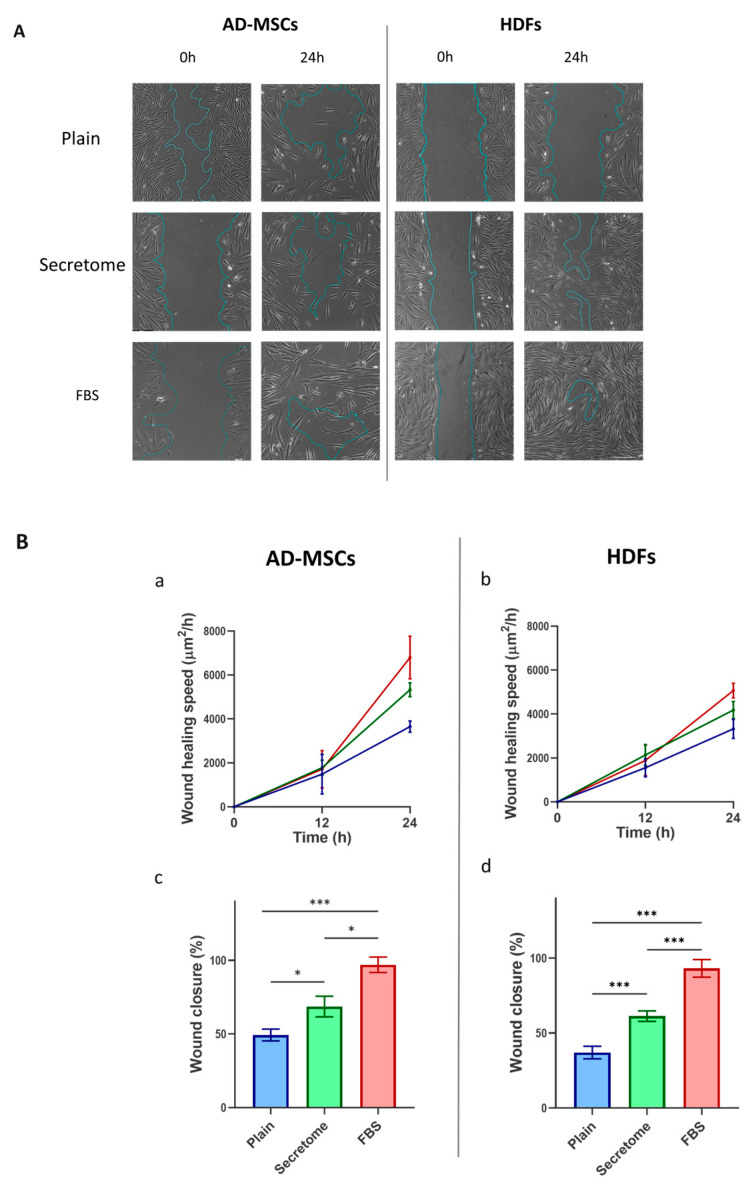
WJ-MSC-derived secretome promotes migration of MSCs and HDFs in an in vitro wound healing assay. Cell migration was evaluated using a scratch assay conducted on collagen-coated substrates over a 24 h period under three culture conditions: plain medium (control), medium supplemented with WJ-MSC-derived secretome (secretome), and medium containing 10% FBS. (**A**) shows representative phase-contrast images of AD-MSCs and HDFs at the start of the assay (left) and after 24 h (right). The initial wound area is marked by a blue line, which also delineates the boundary of the closed region at the endpoint. (**B**) quantifies migration dynamics: graphs (**a**) (AD-MSCs) and (**b**) (HDFs) display migration speed over time, while graphs (**c**) (AD-MSCs) and (**d**) (HDFs) illustrate the percentage of wound closure after 24 h. Data are color-coded: control (blue), secretome-treated (green), and FBS-supplemented (red). Statistical significance is indicated as follows: *p* < 0.05 (*), and *p* < 0.001 (***). Scale bar: 500 μm.

As can be seen in Figure 5, both cell types demonstrated markedly increased migration in response to the WJ-MSC secretome relative to the untreated control, although the extent of migration was lower than that observed under FBS-supplemented conditions. These findings suggest that the WJ-MSC secretome exerts a stimulatory effect on cell motility in both MSCs and HDFs, consistent with a paracrine-mediated pro-migratory mechanism.

## 3. Discussion

Our findings reveal a robust paracrine interaction between Wharton’s WJ-MSCs and adipose-derived AD-MSCs, characterized by enhanced migration, substrate interaction, and extracellular collagen deposition. These effects suggest that MSCs from distinct tissue origins can engage in synergistic signaling, amplifying regenerative outcomes. As specified in the introduction, comparative proteomic analyses have shown that the WJ-MSC secretome possess a broader and more diverse protein profile than those from adult-derived MSCs, including AD-MSCs, with higher levels of immunomodulatory and pro-regenerative factors such as HGF, VEGF, IL-6, and TGF-β1 [17,38]. This molecular richness may underline the superior bioactivity observed in our experiments.

### 3.1. Insights into MSC–Fibroblast Interactions

While fibroblasts also responded to the WJ-MSC secretome, their activation was modest compared to AD-MSCs. This differential responsiveness likely reflects intrinsic differences in cellular plasticity and receptor expression. AD-MSCs, as multipotent progenitors, possess dynamic transcriptional and epigenetic landscapes that allow rapid adaptation to paracrine cues. In contrast, fibroblasts, being more terminally differentiated, exhibit limited transcriptional flexibility and are primarily geared toward ECM maintenance and wound closure. Mechanistic studies have shown that fibroblast activation is tightly regulated by mechanotransduction pathways such as RhoA/ROCK and YAP/TAZ, which respond to substrate stiffness and ECM tension [39]. Moreover, the fibroblast-to-myofibroblast transition (FMT), a hallmark of fibrotic remodeling, is often triggered by persistent TGF-β signaling and mechanical stress. Our data suggests that the WJ-MSC secretome does not induce this transition nor excessive collagen secretion, thus preserving fibroblast quiescence while selectively activating progenitor traits in AD-MSCs.

### 3.2. Substrate Effects on Newly Secreted Collagen Organization

Paracrine stimulation influenced both collagen secretion and its spatial organization within the ECM, underscoring the therapeutic relevance of MSC-derived secretomes in ECM-focused regenerative strategies. The use of species-specific collagen substrates, such as RTC, allowed immunological distinction between endogenous human collagen and exogenous scaffold components, providing a refined platform for studying matrix remodeling. However, under our experimental conditions, RTC appeared suboptimal for supporting the organized fibrillogenesis of newly secreted collagen. Its lack of native crosslinking and nucleation sites, combined with a pre-adsorbed and non-physiological surface topology, likely disrupted the alignment of secreted collagen, resulting in clustered aggregates rather than structured fibrils. Collagen self-assembly is highly sensitive to substrate architecture, as well as to environmental factors like pH and ionic strength [40]. To enhance ECM remodeling and functional integration, future studies should explore advanced 3D scaffolds such as decellularized human matrices or biomimetic hydrogels, which have demonstrated superior support for collagen alignment and structural organization [41].

### 3.3. Dose Considerations for Clinical Translation

The concentration of the secretome applied in vitro does not necessarily reflect its clinical efficacy. Achieving optimal dosing requires a careful balance between biological activity and safety, avoiding both overstimulation and unintended off-target effects. As noted by Gwam et al. (2021) [42], the regenerative potency of MSC-derived secretomes is dose-dependent, with maximal effects typically observed at intermediate concentrations ranging from 50 to 200 µg/mL of the total protein. Higher doses may provoke cellular stress or immune activation, while insufficient concentrations may fail to elicit a meaningful therapeutic response. In our study, we utilized the native concentration of secretomes present in the conditioned medium collected from WJ-MSCs after 48 h of culture (approx. 300 µg/mL total protein) prior to any concentration step, thus reflecting a physiologically relevant exposure level. To ensure reproducibility and translational relevance, the standardization of MSC secretome production is essential. This involves tightly controlling variables such as cell density, culture duration, and the composition of the conditioning medium, all of which significantly influence the secretome’s bioactivity and therapeutic potential [43,44]. Without harmonized protocols, batch-to-batch variability can compromise both experimental outcomes and clinical efficacy, underscoring the need for good manufacturing practice (GMP)-compliant manufacturing strategies [43]. Furthermore, quantitative proteomic profiling and functional bioassays should be employed to define therapeutic windows tailored to specific clinical applications.

### 3.4. Therapeutic Implications of Combinatory MSC Strategies

Our findings support the development of combination MSC therapies that leverage the complementary strengths of different MSC sources. WJ-MSCs offer robust immunomodulatory and proliferative signals, while AD-MSCs contribute lineage-specific differentiation and ECM remodeling. Clinical trials have demonstrated that WJ-MSC-derived exosomes accelerate wound healing and reduce inflammation in diabetic foot ulcers [45], whereas AD-MSCs show promise in cartilage regeneration and osteoarthritis management [46]. A combinatorial approach, either through co-culture, sequential secretome delivery, or engineered hybrid formulations, could enhance therapeutic outcomes by targeting multiple regenerative pathways simultaneously. Furthermore, the ability of the WJ-MSC secretome to activate both the progenitor and stromal cells suggests a dual mechanism of action: recruiting stem cells to sites of injury while mobilizing resident fibroblasts for matrix reorganization. This aligns with emerging paradigms in regenerative medicine that favor cell-free therapies capable of orchestrating endogenous repair processes without the logistical and immunological challenges of cell transplantation, as highlighted by González-González and colleagues (2020) [47].

### 3.5. Immunological Considerations of Allogeneic Secretomes

Although secretome-based therapies are generally considered less immunogenic than whole-cell approaches, the use of allogeneic secretomes still warrants careful immunological evaluation. Donor-derived components—including extracellular vesicles, proteins, and nucleic acids—may retain immunogenic epitopes capable of eliciting host immune responses. This is particularly relevant in allogeneic applications, where inter-donor variability and the absence of standardized secretome profiles may influence immunocompatibility. Emerging strategies to mitigate these risks include preclinical immunogenicity screening, selective depletion of immunostimulatory molecules, and the development of engineered or synthetic secretomes with defined compositions [48]. Furthermore, the route of administration, dosage, and target tissue microenvironment can modulate the host response and should be optimized during translational development. Addressing these immunological aspects is critical to ensure the safety and efficacy of secretome-based interventions in clinical settings.

### 3.6. Study Limitations

Despite the promising in vitro findings, several limitations must be acknowledged to contextualize the translational relevance of this study.

- In vitro constraints: Our experiments were conducted under controlled in vitro conditions, which do not fully replicate the complexity of in vivo tissue environments. Factors such as immune cell interactions, vascularization, mechanical stress, and systemic feedback loops are absent but play critical roles in regenerative outcomes. In vivo validation using relevant animal models is essential to assess therapeutic efficacy, biodistribution, and long-term safety.
- Donor variability: The composition and potency of MSC secretomes are influenced by donor-specific factors including age, sex, metabolic status, and tissue origin. Proteomic profiling has revealed significant inter-donor variability in the abundance of key regenerative proteins such as VEGF, brain-derived neurotrophic factor (BDNF), and the homodimeric isoform of PDGF with 2 “A” chains (PDGF-AA) [49,50]. This underscores the need for standardized donor selection and quality control protocols in secretome production.
- Time-limited assessment: Our analysis focused on cellular responses within a 72 h window, capturing early proliferative, migratory, and ECM remodeling events. However, MSC secretome effects may evolve over longer durations, influencing differentiation, immunomodulation, and tissue integration. Time-course studies extending beyond 72 h are needed to assess sustained activation, potential senescence, or feedback inhibition mechanisms.

## 4. Materials and Methods

### 4.1. Cells

*Human adipose tissue derived mesenchymal stem cells (AD-MSCs)* at passage 2 were obtained from the Tissue Bank BulGen (Sofia, Bulgaria) with informed consent from donors prior to liposuction. Cells were cultured in DMEM/F12 medium (Sigma-Aldrich, St. Louis, MO, USA) supplemented with 10% fetal bovine serum (FBS) (both from Sigma-Aldrich, USA) and 1% antibiotic–antimycotic solution (Sigma-Aldrich, USA) in humidified thermostat at 37 °C, 5% CO_2_. Medium was replaced every 2 to 3 days until cells reached ~90% density and then passaged using 0.05% trypsin/0.6 mM EDTA (Sigma-Aldrich, USA). Cells used for experiments were between passages 5 and 7.

*Primary human dermal fibroblasts (HDFs)* were isolated from fresh skin biopsy also with informed donor consent following the procedure of Ningsih [51]. Briefly, the donor skin was sectioned into approximately 2 × 2 mm fragments, left to attach in a 12-well TC polystyrene plate (Corning, Corning, NY, USA) on an area marked by scratches, which were made to enhance the cell attachment. The explants were cultured in DMEM/F12 medium (Sigma-Aldrich, USA) supplemented with 10% FBS (Sigma-Aldrich, USA) and 1% antibiotic–antimycotic solution (Sigma-Aldrich, USA) in humidified thermostat at standard conditions (37 °C, 5% CO_2_). Once fibroblasts began migrating out of the explant, the culture medium was replaced every 3–5 days. Primary cultures were maintained for approximately 10 days until reaching ~70–80% density. Cells were then harvested with trypsin–EDTA solution (Sigma-Aldrich, USA), and its activity was neutralized by adding an equal volume of FBS, then the cell suspension was centrifuged at 300× *g* for 5 min. The pellet was resuspended in fresh medium and seeded in a T25 TC polystyrene flask (Corning, USA) and left for subsequent cultivation in standard conditions.

### 4.2. Secretome Preparation

Human umbilical cord tissue from healthy donors was obtained with informed consent, approved by the Ethics Committee of Ob/Gyn Hospital Dr. Shterev, Sofia, Bulgaria. Mesenchymal stem cells (MSCs) were isolated and characterized by Tissue Bank BulGen based on the expression of MSC-specific markers and absence of hematopoietic ones. Briefly, umbilical cord segments (2.5 cm) were dissected to remove blood vessels, minced, and enzymatically digested with 0.25% Collagenase I, 4 mg/mL hyaluronidase, and 1% Pen/Strep/A at 37 °C for 3 h in a humidified 5% CO_2_ incubator. The digested tissue was diluted with saline, filtered through a 70 μm strainer, and centrifuged at 876× *g* for 10 min. The cell pellet was re-suspended in DMEM/F12, supplemented with 10% FBS and 1% antibiotic–antimycotic solution (all from Sigma-Aldrich), and then seeded at density 1 × 10^4^ cells per 25 cm^2^ flask. Medium was refreshed every 48 h until cells reached around 80–90% density. At that point, the medium was replaced with fresh serum-free medium and cultured for 48 h before being collected. The collected medium containing secretome was centrifuged at 876× *g* for 10 min (to remove debris) and concentrated 10 times using Amicon Ultra centrifugal filter units (Amicon, Schorndorf, Germany). The protein concentration was measured using Bradford assay (Sigma-Aldrich, USA) and determined to be approx. 0.3 mg/mL. The secretome was then lyophilized and stored at −80 °C until use.

### 4.3. Preparation of Collagen-Coated Surfaces

Collagen type I was produced from rat tail tendon by acetic acid extraction and salting out with NaCl, as described elsewhere [52]. The pellets were collected by centrifugation at 4000 rpm at 4 °C for 30 min and re-dissolved in 0.05 M acetic acid, then dialyzed to remove the excess NaCl. All procedures were performed at 4 °C. The collagen concentration in the solutions was measured by the modified Lowry assay [53] and from the optical absorbance at 220–230 nm.

Collagen-coated 24-well culture plates (Greiner Bio-One, Frickenhausen, Germany) or 22 × 22 mm glass coverslips (ISOLAB GmbH, Frankfurt am Main, Germany, placed in 6-well plates), were coated with 100 µg/mL rat tail collagen (isolated as above) dissolved in 0.05 M acetic acid via incubation at 37 °C for 1 h, before samples were rinsed three times with phosphate-buffered saline (PBS).

### 4.4. Secretome Treatment

AD-MSCs and HDFs were seeded at 1.2 × 10^4^ cells/well in 600 µL of serum-free DMEM/F12 (Sigma-Aldrich, USA) medium with 1% antibiotic–antimycotic solution (Sigma-Aldrich, USA) on collagen-coated 24-well plates or alternatively on coverslips in 6-well plates. After 2 h of incubation (37 °C, 5% CO_2_), WJ-MSCs’ secretome was added in final protein concentration of 0.03 mg/mL without adding FBS. Control groups received only serum-free DMEM/F12 medium as a negative control or DMEM/F12 medium supplemented with 10% FBS as a positive control.

### 4.5. Cell Proliferation Assay

Cell proliferation was assessed at 24, 48, and 72 h of culture. Cells were fixed with 4% paraformaldehyde and stained with Hoechst 33258 (1:2000, Sigma-Aldrich) to visualize cells’ nuclei, then viewed with fluorescence microscopy (Thunder Imager Live Cell, Leica, Wetzlar, Germany) with a 10× objective and counted using the CellProfiler software version 4.2.8 [54]. The doubling time was calculated by using the following formula:

$$Doubling\;Time=\left[T\times \left(ln2\right)\right]/[ln(N_{e}/N_{b})]$$

where $N_{b}$ is the initial cell density, $N_{e}$ is the final cell density, and T is the time passed between the two measurements.

### 4.6. Cell Cycle Analysis

Cell cycle distribution was determined in accordance with the protocol of Vassilis Roukos [55]. Briefly, the fluorescent images were taken on a fluorescent microscope with 10× objective. Then a pipeline provided by the team of Roukos was applied to the image set, which allowed the quantification of the DNA in each nucleus. In accordance with the protocol, after acquiring the fluorescent images, they were fed into the first pipeline which determined the illumination correction function to be used for removing the variance in the background between images. Next, all the images, together with the illumination correction function file, were analyzed using a second pipeline. It consists of the following important steps—(1) applying the illumination function, (2) determining the borders of the cells’ nuclei and selecting them as objects, (3) measuring the total internal intensity of each object, and (4) exporting the information in ‘.csv’ files. These files were remastered manually to fit the standard flow cytometry file format. For each experimental condition, at least 600 nuclei were analyzed. Cell cycle analysis was performed using Floreada.io (https://floreada.io/, accessed on 24 April 2025). This was performed using the flow cytometry analysis tool. The nuclear intensity data in the flow cytometry file format was uploaded to the website, and the standard cell cycle analysis Michael H. Fox algorithm [56], provided by the website, was applied. Nuclei were further classified into 3 groups according to the cell cycle phase (G1/G0, S, and G2/M) and plotted in 3 colored graphs (blue, green, and red, accordingly).

### 4.7. Morphological Analysis and Endogenous Collagen Production

After 24 h of incubation in defined conditions, HDFs and MSCs were fixed in 4% paraformaldehyde and permeabilized with 0.5% Triton X-100 for 5 min before being washed and treated with blocking solution (PBS with 10% FBS (Sigma-Aldrich, USA), 15 min), then incubated (37 °C) with rabbit anti-human collagen 1 (AB745, Sigma-Aldrich, USA) followed by Alexa Fluor^®^ 555-conjugated donkey anti-rabbit IgG (406412, BioLegend, San Diego, CA, USA) as the secondary antibody. All antibodies were applied in 1:100 dilution in PBS containing 10% FBS (Sigma-Aldrich, USA). The actin cytoskeleton was visualized using Alexa Fluor™ 488 phalloidin (Invitrogen, Carlsbad, CA, USA), and the nuclei were counterstained with Hoechst 33258 (1:2000). Fluorescent images were acquired using a fluorescence microscope (Thunder Imager Live Cell, Leica) with 20× objective. In order to determine the overall cell shape, the CellProfiler software [54] was used.

### 4.8. Artificial Wound Healing (Scratch) Assay

MSCs and HDFs were seeded in 12-well plates and incubated until rich around confluent cell density (90%) when a linear scratch was made across the cell monolayer using a sterile 200 µL pipette tip. The samples were studied either plain (medium only) or medium-supplemented with 10% WJ-MSCs secretome (from 10× concentrate) or with 10% FBS as positive control. The cells were incubated using live cell chamber of the inverted microscope (Thunder Imager Live Cell, Leica) in time-laps mode (each 15 min) during 24 h. Images of the wound area were captured at selected time points (0 and 24 h) with 10× objective under phase contrast.

The wound area was quantified using ImageJ software version 1.54p, Wayne Rasband, National Institute of Mental Health, NIH, Bethesda, MD, USA, with a plugin for the high throughput image analysis of in vitro scratch assays [57] and the percentage of wound closure was calculated as follows:

$$\%\;Closure=[(A_{0}-A_{t})/A_{0}]\times 100$$

where $A_{0}$ is the wound area at 0 h and $A_{t}$ is the area at time t.

### 4.9. Statistical Analysis

Statistical analysis was performed using GraphPad Prism 8 (GraphPad Software, San Diego, CA, USA). After the Shapiro–Wilk test for normality was performed, the homogeneity of variances of the data was assessed using Bartlett’s test, which confirmed that the assumption of equal variances was not violated (*p* > 0.05). Then the results were analyzed using one-way analysis of variance (ANOVA) followed by a correction for multiple comparisons using Tukey’s Honest Significant Difference test. Unless otherwise stated, the experimental conditions have been performed in triplicate. Results were displayed as column bar graphs, as scatter plots with each point representing an independent replicate, as line graphs, or as percentage stacked bar charts. Mean ± SEM (or SD) is indicated as described in the figure legends. The confidence limit reflecting the significant difference between experimental groups was considered 95% when *p* < 0.05.

## 5. Conclusions

This study demonstrates that the WJ-MSC-derived secretome selectively activates AD-MSCs while maintaining fibroblast quiescence, highlighting its potential for anti-fibrotic regenerative therapies. The biphasic cell cycle response in AD-MSCs suggests a controlled activation that may preserve stemness and enhance function, contrasting with the static profile of fibroblasts. The use of species-specific collagen substrates enabled a clear distinction between endogenous matrix remodeling and scaffold contributions, offering a methodological advantage for future ECM studies.

Therapeutically, the ability to stimulate AD-MSC proliferation and migration without triggering fibroblast overgrowth supports the development of combination MSC strategies that balance immunomodulation and differentiation while minimizing fibrotic risk. These findings reinforce the promise of cell-free regenerative approaches and call for in vivo validation, donor standardization, and long-term assessment to guide clinical translation.

## Figures and Tables

**Table 1:** Quantitative cell morphology averages of cell spreading area in μm^2^ (CSA) and cell aspect ratio (AR), varying from 1 (for circle) to 0 (for extended line) and characterizing cell polarization.

Condition		Average Cell Spreading Area (CSA) µm^2^	Deviation from the Control of CSA (%)	Average Aspect Ratio (AR)	Deviation from the Control of AR (%)
AD-MSCs	Plain (Control)	4007	-	0.4781	-
	Secretome	5081	26.8	0.548	14.6
	FBS	3389	−15.4	0.3736	−21.9
HDFs	Plain (Control)	971.6	-	0.3825	-
	Secretome	1737	78.8	0.3634	−5.0
	FBS	2289	135.6	0.337	−11.9

## Data Availability

The original contributions presented in this study are included in the article. Further inquiries can be directed to the corresponding author.

## Abbreviations

The following abbreviations are used in this manuscript:

WJ-MSCs	Wharton’s Jelly-Derived Mesenchymal Stem Cells
AD-MSCs	Adipose-Derived Mesenchymal Stem Cells
HDFs	Human Dermal Fibroblasts
RTC	Rat Tail Collagen
FBS	Fetal Bovine Serum
MSCs	Mesenchymal Stem Cells
EVs	Extracellular Vesicles
TGF-β/ SMAD2/3	Transforming Growth Factor-β/Small Body Size Genes and Mothers Against Decapentaplegic Genes 2/3
TGF-β	Transforming Growth Factor β
IL-10	Interleukin 10
HGF	Hepatocyte Growth Factor
VEGF	Vascular Endothelial Growth Factor
IGF-1	Insulin-like Growth Factor 1
FGF-2	Fibroblast Growth Factor 2
IL-6	Interleukin 6
MCP-1	Membrane Cofactor Protein 1
MMPs	Matrix Metalloproteinases
ECM	Extracellular Matrix
PDGF	Platelet-Derived Growth Factor
PBS	Phosphate Buffered Saline
CSA	Cell Spreading Area
AR	Cell Aspect Ratio
FMT	Fibroblast-to-Myofibroblast Transition
GMP	Good Manufacturing Practice
BDNF	Brain-Derived Neurotrophic Factor
PDGF-AA	Homodimeric Isoform of PDGF with 2 “A” Chains
